# Supplementary material for: Antimicrobial Activity the Essential Oil from Croton pluriglandulosus Carn. Leaves against Microorganisms of Clinical Interest
Source: J Fungi (Basel). 2023 Jul 17;9(7):756. doi: 10.3390/jof9070756 (PMC10381380; doi:10.3390/jof9070756)
Supplement: Supplementary file 1 [file jof-09-00756-s001.zip › jof-2414731-supplementary.pdf]

Supplementary Table S1. Main chemical components of *C. pluriglandulosus* essential oil by GC-MS analysis.

	RT	Compounds	Area (%)
1	5.994	*p*-cymene	0.69
2	6.133	eucalyptol (1,8-cineole)	3.75
3	9.101	epoxy-ocimene	0.21
4	10.134	4-terpineol	0.31
5	10.528	α-terpineol	2.65
6	14.611	δ-elemene	2.86
7	15.918	β-elemene	0.17
8	16.115	β-elemene	3.88
9	16.508	methyl eugenol	3.02
10	16.649	1,3,5-trimethoxy-benzene	6.86
11	16.741	α-cis-bergamotene	0.15
12	16.894	caryophyllene	8.99
13	17.251	α-trans-bergamotene	0.87
14	17.675	neryl acetone	0.09
15	17.763	α-humulene	1.44
16	17.953	allo-aromadendrene	0.44
17	18.926	bicyclogermacrene	9.37
18	20.193	hedycaryol	6.21
19	20.547	elemicin	25.77
20	20.977	spathulenol	2.96
21	21.12	epiglobulol	2.17
22	21.329	globulol	0.31
23	21.937	2,4,6-trimethoxy-styrene	3.96
24	22.782	epi-α-muurolol	0.46
25	23.228	brevifolin	4.60
26	23.46	α-bisabolol	0.17
27	24.232	2Z.6Z-farnesol	0.54
28	24.705	2Z.6E-farnesal	0.28
		**Total identified**	**93.18**

RT—Retention time (min); Area (%)—Relative area percentage.
